# Randomized, placebo-controlled, calcium supplementation trial in pregnant Gambian women accustomed to a low calcium intake: effects on maternal blood pressure and infant growth^1^,^2^,^3^,^4^

**DOI:** 10.3945/ajcn.113.059923

**Published:** 2013-09-04

**Authors:** Gail R Goldberg, Landing MA Jarjou, Tim J Cole, Ann Prentice

**Affiliations:** 1 From the Medical Research Council Human Nutrition Research, Cambridge, United Kingdom (GRG and AP); the Medical Research Council, Keneba, The Gambia (GRG, LMAJ, and AP); and the Medical Research Council Centre of Epidemiology for Child Health, University College London Institute of Child Health, London, United Kingdom (TJC).

## Abstract

**Background:** Dietary calcium intake in rural Gambian women is very low (∼350 mg/d) compared with international recommendations. Studies have suggested that calcium supplementation of women receiving low-calcium diets significantly reduces risk of pregnancy hypertension.

**Objective:** We tested the effects on blood pressure (BP) of calcium carbonate supplementation (1500 mg Ca/d) in pregnant, rural Gambian women.

**Design:** The study was a randomized, double-blind, parallel, placebo-controlled supplementation trial from 20 wk of gestation (P20) until delivery (calcium: *n* = 330; placebo; *n* = 332). BP and anthropometric measures were taken at P20 and then 4 weekly until 36 wk of gestation (P36), and infant anthropometric measures were taken at 2, 13, and 52 wk postdelivery.

**Results:** A total of 525 (calcium: *n* = 260; placebo: *n* = 265) women had BP measured at P36 and subsequently delivered a healthy term singleton infant. Mean compliance was 97%, and urinary calcium measures confirmed the group allocation. At P20, the mean (±SD) systolic blood pressure (SBP) was 101.2 ± 9.0 and 102.1 ± 9.3 mm Hg, and diastolic blood pressure (DBP) was 54.5 ± 7.3 and 55.8 ± 7.8 mm Hg, in the calcium and placebo groups, respectively. The intention-to-treat analysis that was adjusted for confounders showed no significant effect of calcium supplementation on the change between P20 and P36 (calcium compared with placebo; mean ± SEM) in SBP (−0.64 ± 0.65%; *P* = 0.3) or DBP (−0.22 ± 1.15%; *P* = 0.8). There was no significant effect of supplementation on BP, pregnancy weight gain, weight postpartum, or infant weight, length, and other measures of growth. However, the comparability of the original randomly assigned groups may have been compromised by the exclusion of 20.7% of women from the final analysis.

**Conclusions:** Calcium supplementation did not affect BP in pregnancy. This result may have been because the Gambian women were adapted to a low dietary calcium intake, and/or obesity, high gestational weight gain, high underlying BP, tobacco use, alcohol consumption, and sedentary lifestyles were rare. This trial was registered at the International Standard Randomized Controlled Trial Register (www.controlled-trials.com/mrct/) as ISRCTN96502494.

## INTRODUCTION

Worldwide, pregnancy complicated by hypertension (estimated to affect 5% of all pregnancies and 11% of first pregnancies) is associated with considerable maternal and infant morbidity and mortality in developed and developing countries (1–3).

The causing factors involved in pregnancy-induced hypertension (PIH)^5^ and preeclampsia are unknown. Maternal diet (intakes of energy, macronutrients, minerals, and antioxidant vitamins), the potential mechanisms that underlie associations between maternal dietary exposures and preeclampsia risk, and the findings of epidemiologic and intervention studies have been extensively reviewed (4). Calcium has been a particular focus of attention (2, 5–18). A Cochrane review published in 2010 concluded that the greatest reductions in risk with supplementation were in women with the lowest calcium intakes [eg, an effect size of 0.44 risk reduction for high blood pressure (BP) for women on a low-calcium diet] (2).

The habitual dietary calcium intake in rural Gambian women is extremely low (typically 300–400 mg Ca/d) (19) compared with international recommendations that range from 700 to 1000 mg Ca/d (20–22). National studies have shown a prevalence of hypertension of 9% in The Gambia in males and females >15 y old (23–25). Maternal mortality and morbidity are also high (26, 27). The Gambia Maternal Mortality Survey indicated that, for every 680 live births in The Gambia, one pregnant mother died of eclampsia (27). In the West Kiang region, raised BP (>140/90 mm Hg) was diagnosed in ∼3% of pregnancies (28). The low birth weight and poor growth of infants in rural areas of The Gambia and the effects of season are well documented (29, 30).

The aims of this trial were to test, in a double-blind, randomized, parallel, placebo-controlled, calcium-supplementation trial, whether an increase in calcium intake in the second half of pregnancy significantly and beneficially affects maternal BP in late pregnancy in Gambian women and infant growth. The trial was registered on the International Standard Randomized Controlled Trial Register (http://www.controlled-trials.com/mrct/) as ISRCTN96502494. The primary outcome measure of the trial was maternal BP at 36 wk of gestation. Secondary outcomes were maternal BP during the first year postpartum and infant growth ≤1 y of age. The breast-milk calcium concentration, infant growth, and bone mineral content ≤1 y of age and maternal dietary calcium intake and bone and biochemical outcomes have already been reported in a subset studied in more detail (31, 32).

## SUBJECTS AND METHODS

The research was conducted at Medical Research Council (MRC) Keneba, where a clinic has been run for the local population for many years (30), and in 2 outreach clinics. The trial was approved by The Gambia Government/MRC Joint Ethics Committee. Written informed consent was obtained from all participants after an oral explanation was given in the local language.

### Sample size and statistical power

The trial was designed to investigate the effects of calcium supplementation on BP in pregnancy. Previous studies conducted by other authors (5, 6, 10) had shown differences in systolic blood pressure (SBP) of 4–10 mm Hg and diastolic blood pressure (DBP) of 3–8 mm Hg at 36–38 wk of gestation between unsupplemented and supplemented women. For this trial, typical between-individual CVs in the Gambian population measured in the MRC Keneba clinic for SBP (10%) and DBP (12%) were used. At 5% significance and 80% power, a sample size of 260 subjects/group was estimated to enable minimum detectable between-group differences at 36 wk of gestation in SDP and DBP of 2.4% (equivalent to 2.5 mm Hg when the mean is 105 mm Hg) and 2.9% (equivalent to 2.0 mm Hg when the mean is 68 mm Hg), respectively. These differences are physiologically plausible, and if observed, would show a biologically significant effect of calcium supplementation.

### Participant recruitment and trial design

The aim was to recruit ≥600 women to allow for dropouts and have a final data set that comprised ∼260 participants/group. The trial was conducted in 16 rural villages in the province of West Kiang, The Gambia. Potential participants were pregnant women with no medical history that affected calcium metabolism and who presented for booking at the antenatal clinic at the MRC Keneba (clinic 1) or outreach antenatal clinics held throughout West Kiang (clinics 2 and 3). All women who attended clinics during the period of the trial (recruitment began in May 1995 and ended in March 2000) were considered for recruitment. The women were invited to participate if they were identified by the clinic midwife as having an uncomplicated singleton pregnancy, lived locally, were unlikely to be away from home for prolonged periods, and were between 18–22 wk of gestation estimated by fundal height. No records were kept of numbers or details of women considered eligible who were invited but declined to participate.

Women who apparently met the inclusion criteria and agreed to take part were randomly assigned to the calcium or placebo group and attended village antenatal clinics at 20 wk of gestation (P20) for baseline measurements of BP, weight, and height. The same measurements were performed at the clinics by trained field staff during usual antenatal care at 24 (P24), 28 (P28), 32 (P32), and 36 (P36) wk of gestation. Visits were made at intervals of 4.4 ± 1.7 (P20–P24), 4.1 ± 0.6 (P24–P28), 4.2 ± 0.6 (P28–P32), and 3.5 ± 0.5 (P32–P36) wk in the calcium group and 4.0 ± 1.5 (P20–P24), 4.2 ± 0.6 (P24– P28), 4.2 ± 0.6 (P28–P32), and 3.5 ± 0.6 (P32–P36) wk in the placebo group. The trial protocol required that any participant who developed complications such as preeclampsia would be treated. The protocol also specified exclusions on the grounds of death or incorrect gestation after random assignment and not post hoc.

Postpartum, in line with the standard practice at the MRC Keneba, field workers and medical staff visited the mother at home when possible during the first week after delivery to record infant weight, length, and head circumference. Gestational age was assessed by using a modified scoring system of Dubowitz et al (33). Thereafter, for this trial, mothers (for BP and anthropometric measures) and infants (for anthropometric measures) were visited at 2 wk postdelivery (calcium group: 2.1 ± 1.2 wk; placebo group: 2.1 ± 1.2 wk), 13 wk postdelivery (calcium group: 13.7 ± 1.8 wk; placebo group: 13.6 ± 1.3 wk), and 52 wk postdelivery (calcium group: 52.8 ± 1.3 wk; placebo group: 53.0 ± 1.5 wk).

### Calcium supplementation

The 3 antenatal clinics each covered a cluster of villages in different geographical regions of West Kiang. Random assignment was stratified by clinic to take these clusters of villages into account and, thereby, minimize the potential for bias because of differences in antenatal care. Participants were randomly allocated, double-blinded, and stratified by antenatal clinic to receive a calcium supplement or placebo from P20 until delivery. The assignment within each stratum was by random permuted blocks of 4 to ensure that equal numbers of participants were allocated to calcium and placebo groups each week at each clinic and, thereby, minimize the potential for seasonal confounding. The allocation sequence was generated by using random-number tables. The code was held by a member of the trial team (AP) who was not directly involved with data collection and had no contact with participants.

The calcium supplement provided 1500 mg elemental Ca/d and consisted of 3 chewable calcium carbonate tablets (Calcichew; Nycomed Pharma AS; distributed in the United Kingdom by Shire Pharmaceutical Development Ltd), each of which contained 500 mg elemental Ca. The placebo consisted of 3 tablets of similar shape, taste, and texture in which the calcium carbonate was replaced with microcrystalline cellulose and lactose (Nycomed Pharma AS). Each participant started to receive their tablets after P20 measurements were completed and continued until delivery.

Tablets were consumed between 1700 and 1900 (ie, the period between lunch and dinner) to minimize a possible interference with the absorption of other minerals. Tablets were consumed later during the fast month of Ramadan, immediately after participants had broken their fast but before the main meal. Participants who attended clinic 1, which included all subjects in the detailed subset (31, 32), were given their tablets by field staff each day, and tablets were consumed in the presence of the field staff. The intake of the tablets was noted each day by the field staff as a marker of compliance. Tablets not consumed because of illness or absence were counted as missed doses. In clinics 2 and 3, participants were given color-coded tickets printed with their trial identification every 2 wk. Subjects were invited to make daily early evening visits to their local traditional birth attendant with whom individuals’ jars of tablets, appropriately color-coded, were lodged and to exchange a ticket for 3 tablets, which participants consumed in the presence of the traditional birth attendant. Compliance was monitored regularly by a ticket and tablet count by a member of the trial team, and reasons for missed doses were noted.

## MEASUREMENTS

### BP

SDP and DBP (fifth Korotkoff sound) were measured by using an automated sphygmomanometer (Dinamap 8100; Critikon Ltd UK) as used previously at the MRC Keneba (34) and in large national surveys in the United Kingdom [eg, Finch et al (35)].

The protocol was highly standardized. Measurements were made in a quiet environment by trained field staff. The participant was seated at rest for 5 min beforehand and was asked to confirm that she had not taken tobacco or kola nut for ≥2 h. A cuff of appropriate size [which was based on the midupper arm circumference (MUAC)] was placed on the right arm at the level of the heart, and the same cuff was used for each individual at all visits. The participant was seated on a chair sideways to the bench or table that the BP monitor was on. The arm of the subjects rested comfortably on the table with the elbow bent, upper arm straight, and lower arm at a right angle with the palm facing upwards. The elbow was at the level of the heart. Sleeves were rolled up, or constricting clothing was removed. The participants sat with legs uncrossed and both feet flat on the ground or a footrest. Measurements were made according to the manufacturer's instructions. The procedure was explained to the participant so that they would not be alarmed when the cuff inflated automatically. Three measurements were made at 2-min intervals.

### Anthropometric measures

Measurements were made by trained field staff by using standard protocols, and all equipment was regularly checked with known weights and length measures. Maternal weight was measured to the nearest 0.1 kg while the subjects wore light clothing and no shoes (Wylux digital scales; CMS Weighing Equipment Ltd). Maternal standing height without headwear or shoes was measured under standardized conditions (horizontal Frankfort plane) recorded to the nearest 0.1 cm (Magnimetre stadiometer; CMS Weighing Equipment Ltd). Height was measured on each occasion during the trial, and the mean of measurements was used.

Infants were weighed naked to the nearest 0.01 kg (Seca baby-weighing scale; CMS Weighing Equipment Ltd). Supine crown-heel length was measured with a length board (Kiddimate; Raven Equipment Ltd). Head circumference and MUAC were measured by using a nonstretchable tape. Triceps skinfold thickness was measured by using Holtain calipers (Holtain Ltd).

### Maternal urine samples

Maternal urine samples were collected at P20 and P36 for later analysis of urinary calcium to confirm the correct allocation to the group. As previously described, women who were studied in detail provided 24-h urine samples under standardized conditions (31, 32). The other participants provided a spot sample from a midstream urine collected for dipstick analysis as part of the usual antenatal care. Urinary calcium and creatinine concentrations were analyzed as described previously (31, 32).

### Statistical analysis

Descriptive statistics are presented as means ± SDs, and differences were presented as means ± SEMs, unless otherwise stated. Urinary calcium data were positively skewed and are presented as the geometric mean (geometric mean −1 SD; geometric mean +1 SD). The statistical analysis was performed by using Student's *t* test, ANOVA, ANCOVA, logistic regression (for parity data), and conditional multiple regression analysis with Linear Model software in the DataDesk version 6.3.1 program (Data Description Inc). Data were transformed into natural logarithms to allow the investigation of power relations between continuous variables and proportional (percentage) effects of discrete variables. When the dependent variable is in ln, the regression coefficient for a discrete variable, once multiplied by 100, corresponds closely to the percentage effect as defined as follows (36):


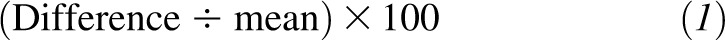


All percentage differences reported in this article were derived in this way. In all cases, the distribution of log-transformed variables approximated normality.

The first BP measurements recorded at each time point were consistently higher, and thus, the mean of the second and third measurements was used for the analysis. However, the outcome of the supplementation trial on BP was not materially different if all 3 measures were used.

The Gambia has distinct seasons, which are usually defined as wet (July to December) and dry (January to June), which influence adult body habitus, pregnancy weight gain, birth weight, and child growth (29, 30, 37). When we examined the data in this trial, it was apparent that the clearest discriminator of effects of the time of year on absolute values and changes in maternal weight was a breakdown into cold (November to February), hot (March to June), and wet (July to October). Because weight is such a strong predictor of BP, these 3 seasons were included in all relevant models. Birth order has been shown to affect infant growth in The Gambia (38). As in other populations (39), nulliparous women are considered to be at higher risk of gestational hypertension. Therefore, all models of maternal and infant data were constructed to include season and parity (nulliparous compared with parous) as confounders, and interactions between group and parity or season were considered as appropriate. Maternal age was not included in any of the models because data available from clinics 2 and 3 were incomplete, and parity is closely related to age in this population (40).

#### Confounding variables

To identify potential confounders in the effects of calcium supplementation on BP and anthropometric measures, significant predictors of SBP, DBP, and weight at P20 and P36 were identified by constructing models that included the following independent variables: parity (nulliparous compared with parous), height, season of measurement (cold, hot, or wet), calendar year, clinic (1, 2, or 3), week of gestation (calculated from the difference between the date of the measurement and the actual date of delivery), and, for SBP and DBP only, weight.

#### Primary outcome

The effect of the calcium supplement on maternal SDP and DBP at P36 was analyzed by using an intention-to-treat (ITT) analysis. Multiple regression models were constructed with SBP or DBP at P36 as the dependent variable. Independent variables were the group (calcium compared with placebo), value of the dependent variable at P20 (to look at the change within individuals and adjust for the regression to the mean), mean of maternal weight at P20 and P36, change in maternal weight between P20 and P36, and all relevant potential confounders previously listed. Nonsignificant independent variables (*P* > 0.05) were removed by backward elimination to produce a final parsimonious model. An as-treated (per protocol) analysis was also conducted that related the number of tablets a woman consumed to her BP at P36. The following 2 nonrandomized observational variables represented compliance: the total number of tablets consumed and duration of supplementation. The effect of the total calcium dose consumed was considered by including an interaction term between compliance and the supplement group.

#### Secondary outcomes

Secondary outcomes in the mother were weight gain by P36, BP, and weight change at 2, 13, and 52 wk postpartum. Multiple regression models with all potential confounders as previously described were constructed for SBP, DBP, or weight at the relevant time point as the dependent variable, and parsimonious models were obtained.

Secondary outcomes in infants were weight, length, triceps skinfold thickness, MUAC, and head circumference at birth and 2, 13, and 52 wk of age. ANOVA or ANCOVA with Scheffe's post hoc tests were used to determine differences according to maternal calcium or placebo groups. Effects on growth velocity were tested by using repeated-measures ANOVA, or ANCOVA as appropriate, performed with the use of hierarchical linear models that included a participant identifier and sex (nested by group), time point, and birth order (parity of the mother; nulliparous compared with parous). An interaction term (group-by-time point) was included to consider the possible effect of maternal calcium supplementation on the rate of change in the dependent variable over time.

## RESULTS

**FIGURE 1.**
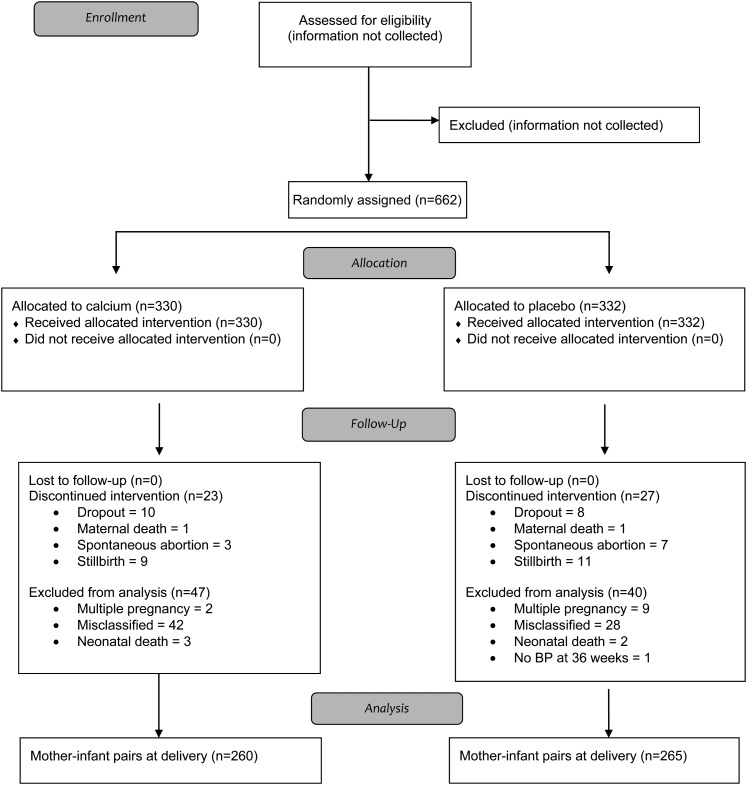
Flowchart of recruitment, exclusions, and losses for the trial Calcium requirements of pregnant women in The Gambia (http://www.controlled-trials.com/mrct/; ISRCTN96502494). For participant data excluded from analysis because of misclassification or multiple pregnancy, misclassified refers to women who were more advanced in gestation at enrollment than diagnosed by the midwife and, therefore, delivered a term baby earlier than anticipated; multiple pregnancy refers to one not identified as such at recruitment by the midwife. This classification became apparent later during pregnancy or at delivery. BP, blood pressure.

In total, 662 women were enrolled and randomly assigned to either the calcium group (*n* = 330) or placebo group (*n* = 332). The flow of participants through the trial is described in **Figure 1** in accordance with the Consolidated Standards of Reporting Trials (41). Exclusions and discontinuations after enrollment and random assignment (*n* = 70 in the calcium group; *n* = 67 in the placebo group) were because of maternal, fetal, neonatal, or infant death; later evidence of an underestimation of the week of gestation at enrollment (ie, women who were more advanced in gestation than diagnosed by the midwife and who, therefore, delivered a term baby earlier than anticipated) or a multiple pregnancy missed at recruitment by the midwife; and missing the P36 BP measurement, moving away, or withdrawing from the trial. No participant was excluded because of pregnancy complications, including clinical hypertension and preeclampsia.

Participant characteristics are shown in **Table 1**. At enrollment, there were no significant differences between calcium and placebo groups. Participants who dropped out or whose data were excluded from the final data set (*n* = 137) had significantly higher SBP (*P* = 0.02) and DBP (*P* = 0.04), tended to have higher weight (*P* = 0.06), and were more likely to be nulliparous (27% of discontinued subjects were nulliparous compared with 14% in subjects who remained in the trial; *P* = 0.0006). There were no significant differences in the season of enrollment (*P* = 0.6) or numbers allocated to or differences in other characteristics between calcium and placebo groups (*P* > 0.1).

**TABLE 1:** Characteristics of participants in the trial*^1^*

	Enrolled into trial (*n* = 662)		Excluded and discontinued (*n* = 137)*^2^*		Included in final data set (*n* = 525)*^3^*	
	Calcium group (*n* = 330)	Placebo group (*n* = 332)	Calcium group (*n* = 70)	Placebo group (*n* = 67)	Calcium group (*n* = 260)	Placebo group (*n* = 265)
Age (y)*^4^*,*^5^*	26.8 ± 6.9	27.2 ± 7.2	24.2 ± 6.8	25.2 ± 7.5	27.4 ± 6.8	27.4 ± 7.2
Weight (kg)*^4^*	55.3 ± 7.4*^6^*	55.2 ± 7.4*^6^*	56.3 ± 9.2	56.4 ± 8.5	55.1 ± 6.9*^7^*	54.9 ± 7.1*^7^*
Height (m)*^4^*,*^8^*	1.60 ± 0.05	1.60 ± 0.06	1.60 ± 0.05	1.60 ± 0.06	1.60 ± 0.05	1.60 ± 0.06
BMI (kg/m^2^)*^4^*	21.6 ± 2.5	21.5 ± 2.5	21.8 ± 3.2	21.9 ± 2.4	21.5 ± 2.3	21.4 ± 2.5
SBP (mm Hg)*^4^*	101.8 ± 9.5	102.3 ± 9.6	104.4 ± 12.3	103.2 ± 10.7	101.2 ± 8.5	102.1 ± 9.3
DBP (mm Hg)*^4^*	54.9 ± 7.7	56.0 ± 7.9	56.3 ± 9.0	57.0 ± 8.3	54.5 ± 7.3*	55.8 ± 7.8
Weeks of gestation*^9^*	—	—	—	—	20.5 ± 3.0	20.2 ± 3.0
Parity*^10^*,*^11^*	3 (1, 6)	3.5 (1, 6)	3 (0.5, 5)	2 (0, 6)	3 (1.75, 6)	3 (1, 6)
Nulliparous [*n* (%)]	53 (16)	56 (17)	17 (25)	19 (29)	36 (14)	37 (14)
Primiparous [*n* (%)]	35 (11)	40 (12)	8 (12)	11 (17)	27 (11)	29 (11)
Parity 2–5 [*n* (%)]	144 (44)	135 (41)	28 (41)	15 (23)	116 (46)	120 (46)
Parity 6–9 [*n* (%)]	84 (25)	88 (26)	14 (20)	19 (29)	70 (28)	69 (26)
Parity 10+ [*n* (%)]	5 (1)	7 (2)	1 (1)	1 (1)	4 (2)	6 (2)
P20 season [*n* (%)]*^12^*						
Cold	84 (25)	89 (27)	16 (23)	21 (31)	68 (26)	68 (26)
Hot	115 (35)	113 (34)	20 (28)	25 (38)	95 (37)	88 (33)
Wet	131 (40)	130 (39)	34 (49)	21 (31)	97 (37)	109 (41)

1 All differences between calcium and placebo groups were NS (*P* > 0.1) except for DBP (*P = 0.05). At enrollment, there were no significant differences between calcium and placebo groups. Participants who dropped out or whose data were excluded from the final data set (*n* = 137) had significantly higher SBP (*P* = 0.02), DBP (*P* = 0.04), tended to have higher weight (*P* = 0.06), and were more likely to be nulliparous (27% of discontinued were nulliparous compared with 14% in those remaining in the trial; *P* = 0.0006). There were no significant differences in the season of enrollment (*P* = 0.6) or numbers allocated to, or differences in, other characteristics between calcium and placebo groups (*P* > 0.1). Statistical tests included Student's *t* test, ANOVA, ANCOVA, logistic regression (for parity data), and conditional multiple regression analysis performed with Linear Model software in the DataDesk version 6.3.1 program (Data Description Inc). DBP, diastolic blood pressure; P20, 20 wk of gestation; SBP, systolic blood pressure.

2 Because of maternal, fetal, neonatal, or infant death, later evidence of underestimation of the week of gestation at enrollment (ie, women who were more advanced in gestation than diagnosed by the midwife and who delivered a term baby earlier than anticipated) or carrying a multiple pregnancy missed at recruitment by the midwife, missing the BP measurement at 36 wk of gestation, missing the blood pressure measurement at 36 wk of gestation, moving away, or withdrawal.

3 Gestation of 18–22 wk at the start of the intervention with a blood pressure measurement at 36 wk of gestation and a healthy singleton term infant.

4 Mean ± SD (all such values).

5 *n* = 201/330, 193/332, 68/70, 65/67, 167/260, 166/265.

6 *n* = 329 in calcium group and *n* = 329 in placebo group.

7 *n* = 259 in calcium group and *n* = 263 in placebo group.

8 Mean of 8 measurements made during the trial.

9 Calculated as difference between date of baseline measurement and date of delivery.

10 All values are medians; IQRs in parentheses.

11 *n* = 321/330, 326/332, 34/70, 27/67, 253/260, 261/265.

12 Cold season: November to February; hot season: March to June; wet season: July to October.

The data set for analysis comprised 525 women (*n* = 260 in the calcium group; *n* = 265 in the placebo group). BP and weight data in pregnancy are shown in **Table 2**. There were no significant differences in age, height, weight, BMI (in kg/m^2^), parity, season, weeks of gestation, time intervals between measurements, or SBP between calcium and placebo groups at P20 (all *P* > 0.1). DBP was slightly lower in the calcium group at baseline in those women who had BP measured at P36 (calcium-placebo mean ± SE: 1.3 ± 0.6 mm Hg; *P* = 0.04).

**TABLE 2:** Maternal blood pressure and body weight during pregnancy*^1^*

Measurement and time point	Calcium group*^2^*	*n*	Placebo group*^2^*	*n*	Calcium compared with placebo*^3^*	*P*
SBP (mm Hg)						
P20	101.2 ± 9.0	260	102.1 ± 9.3	265	−0.9 ± 0.8	0.2
P24	100.0 ± 8.9	243	100.7 ± 9.0	246	−0.7 ± 0.8	0.4
P28	99.6 ± 8.2	235	100.8 ± 9.0	242	−1.2 ± 0.8	0.1
P32	100.9 ± 8.7	233	101.9 ± 8.9	243	−1.08 ± 0.8	0.2
P36	103.4 ± 8.9	260	105.0 ± 10.3	265	−1.6 ± 0.8	0.1
ΔP20–P36	+2.2 ± 8.4*^4^*	260	+2.8 ± 9.3*^4^*	265	−0.6 ± 0.8	0.4
DBP (mm Hg)						
P20	54.5 ± 7.3	260	55.8 ± 7.8	265	−1.3 ± 0.7	0.04
P24	53.8 ± 7.5	243	54.3 ± 7.2	246	−0.5 ± 0.7	0.4
P28	53.9 ± 6.5	235	54.5 ± 7.8	241	−0.6 ± 0.7	0.3
P32	55.2 ± 7.3	233	56.1 ± 7.7	243	−0.9 ± 0.7	0.2
P36	58.4 ± 8.2	260	59.6 ± 9.3	265	−1.2 ± 0.8	0.1
ΔP20–P36	+4.0 ± 8.8*^4^*	260	+3.8 ± 9.1*^4^*	265	−0.2 ± 0.8	0.8
Weight (kg)						
P20	55.1 ± 6.9	259	54.9 ± 7.1	263	0.2 ± 0.6	0.7
P24	56.6 ± 7.0	244	56.4 ± 7.2	244	0.2 ± 0.6	0.7
P28	57.6 ± 7.0	235	57.6 ± 7.5	241	0.1 ± 0.6	0.9
P32	58.4 ± 7.1	237	58.0 ± 7.2	245	0.4 ± 0.7	0.5
P36	59.2 ± 7.3	259	59.2 ± 7.6	265	−0.1 ± 0.6	0.8
ΔP20–P36	+4.0 ± 2.5*^4^*	258	+4.3 ± 2.7*^4^*	263	−0.3 ± 0.2	0.2

1 Differences between calcium and placebo groups at P36 are expressed as percentages derived from regression analysis with adjustment for values at P20. Statistical analysis was performed by using conditional multiple regression analysis with Linear Model software in the DataDesk version 6.3.1 program (Data Description Inc). P#, no. of weeks of gestation.

2 All values are means ± SDs.

3 All values are differences ± SEMs.

4 Significantly different within-group changes between P20 and P36 (all *P* < 0.0001; paired *t* test).

The duration of supplementation was similar in the 2 groups (calcium: 20.5 ± 3.0 wk; placebo: 20.2 ± 3.0 wk; *P* = 0.3). The tablets were well accepted, no adverse effects were reported, and tablet compliance was high. The mean (±SD) number of tablets consumed was 412 ± 61 and 414 ± 60 in calcium and placebo groups, respectively. The mean (±SD) compliance (as a percentage of tablets allocated) was 97 ± 6% in the calcium group and 97 ± 4% in the placebo group.

Urinary calcium (mmol/L) and creatinine (mmol/L) concentrations were measured in 492 samples at P20 (94% of subjects) and in 473 samples at P36 (90%). At P20, in 24-h urine samples, the geometric mean (−1 SD, +1 SD) urinary calcium:creatinine ratio (mmol/mmol) was 0.088 (0.041, 0.191) in the calcium group and 0.084 (0.035, 0.203) in the placebo group (*P* = 0.7). In spot-urine samples, corresponding values were 0.154 (0.065, 0.362) and 0.172 (0.064, 0.463) (*P* = 0.2). As anticipated, calcium supplementation had a significant effect at P36. In 24-h samples, the urinary calcium:creatinine ratio was 0.129 (0.064, 0.259) in the calcium group and 0.075 (0.034, 0.163) in the placebo group (*P* = 0.003). In spot samples, values were 0.221 (0.585, 0.083) and 0.189 (0.472, 0.076), respectively (*P* = 0.1). The mean (±SEM) percentage of change in the urinary calcium:creatinine ratio between P20 and P36 in 24-h samples was +38 ± 11% and −14 ± 13% in calcium and placebo groups, respectively (both *P* = 0.0001); a difference of 54 ± 13% in the change adjusted for the ratio at P20 (*P* = 0.0002). In spot samples, the change between P20 and P36 was +31 ± 9% (*P* = 0.0005) and 9 ± 10% (*P* = 0.4), respectively; which was a difference of 15% in the change adjusted for the ratio at P20, which was not significant (*P* = 0.1). When 24-h and spot samples were combined and adjusted for sample type, there was an increase in urinary calcium:creatinine ratio of 25 ± 8% (*P* = 0.002).

At both P20 and P36, weight differed by the clinic area (*P* = 0.009 at P20 and *P* = 0.05 at P36). Weight was not significantly related to the current season at P20 (*P* = 0.3) but was related at P36 [*P* = 0.005 (hot greater than wet, *P* = 0.01; hot greater than cold; *P* = 0.02). Weight increased from P20 to P36 (calcium: +4.0 kg; placebo: +4.3 kg), but there were no differences between groups (Table 2).

Significant independent positive predictors of both SBP and DBP at P20 were current weight (both *P* < 0.0001) and current season (*P* = 0.002 for SBP and *P* < 0.0001 for DBP). In addition, nulliparity and the week of gestation were significant positive predictors of SBP (*P* < 0.0001 and *P* = 0.03). There were no parity-by-season interactions.

At P36, current weight and nulliparity (both *P* < 0.0001), but not the current season, were significant predictors of SBP. Significant predictors of DBP were nulliparity (*P* < 0.0002), current weight (*P* < 0.0001), and current season (*P* = 0.006; both cold and hot greater than wet).

SBP (calcium: +2.2 mm Hg; placebo: +2.8 mm Hg) and DBP (calcium: +4.0 mm Hg; placebo: +3.8 mm Hg) increased from P20 to P36, but there were no significant differences between groups (Table 2).

ITT models showed no significant difference between calcium and placebo groups in the change in SBP at P36 compared with P20 (mean ± SEM percentage of change: −0.64 ± 0.65 mm Hg; *P* = 0.3), DBP (mean ± SEM percentage of change: −0.22 ± 1.15 mm Hg; *P* = 0.8), or body weight (mean ± SEM percentage of change: −0.38 ± 0.38 kg; *P* = 0.3). Predictors (all positive) that remained significant in the final parsimonious models were, for SBP, change in weight (*P* = 0.002), mean weight (*P* = 0.001), nulliparity (*P* < 0.0001), and season at P20 [*P* = 0.001 (wet greater than cold, *P* = 0.01; wet greater than hot, *P* = 0.004)]; for DBP, mean weight (*P* = 0.003), nulliparity (*P* = 0.0001), season at P20 [*P* < 0.0001 (wet greater than cold, *P* = 0.03; wet greater than hot, *P* = 0.0001)]. For weight, significant positive predictors were height (*P* < 0.0001) and season [*P* < 0.0001 (hot < cold and hot > wet both *P* < 0.0001)].

There were significant increases between P20 and P36 in SBP, DBP, and weight in both calcium and placebo groups (all *P* < 0.0001). Except for the small difference in DBP at P20, there were no significant differences in SBP, DBP, or weight between calcium and placebo groups (Table 2).

One woman had a P36 DBP >90 mm Hg (92.5 mm Hg in the placebo group). Two women (0.4%) had increases of SBP >30 mm Hg (49.5 mm Hg in the calcium group; 44.5 mm Hg in the placebo group). Increases in DBP >15 mm Hg (range: 15.5–33.0 mm Hg) were measured in 54 women (calcium group: *n* = 24; placebo group: *n* = 30; including the woman with DBP >90 mm Hg). There was no difference between calcium and placebo groups (chi-square test *P* = 0.52).

In the as-treated analysis, there was no significant difference between calcium and placebo groups in the change between P20 and P36 in SBP (calcium-placebo mean ± SEM percentage of change: −0.44 ± 0.63 mm Hg; *P* = 0.5), DBP (mean ± SEM percentage of change: −0.00 ± 1.1 mm Hg; *P* = 0.9), or weight (mean ± SEM percentage of change: −0.45 ± 0.38 kg; *P* = 0.2). The number of tablets consumed and duration of supplementation were not significantly different, and there were no significant compliance-by-supplement group interactions.

Of 525 infants born to mothers in the trial, 270 infants were girls (132 and 138 girls in calcium and placebo groups respectively), and 255 infants were boys (128 and 127 boys in calcium and placebo groups, respectively). Many mothers spent the traditional 8-d confinement period away from their village, and neonatal assessments could not be made in 165 infants (*n* = 88 and *n* = 77 for calcium and placebo groups, respectively). Infant anthropometric data according to the group allocation of mothers are shown in **Table 3**. There were no significant differences at birth between groups in gestational age, weight, length, and head circumference or in weight, length, head circumference, MUAC, or triceps skinfold thickness at 2, 13, or 52 wk of age. As expected, these measures increased significantly with infant age, and there were significant differences between boys and girls. There were no interactions with the supplement group allocation of the mother.

**TABLE 3:** Infant anthropometric measurements by group allocation of mothers*^1^*

Time point and measurement	Calcium group*^2^*	*n*	Placebo group*^2^*	*n*	Calcium compared with placebo*^3^*	*P*
Birth*^4^*						
Gestational age (wk)	38.3 ± 1.5	172	38.2 ± 1.5	186	0.02 ± 0.16	0.9
Length (mm)	485 ± 24	171	487 ± 26	187	−1.5 ± 2.7	0.6
Weight (kg)	2.9 ± 0.4	171	2.9 ± 0.4	188	−0.04 ± 0.04	0.3
Head circumference (mm)	339 ± 12	172	340 ± 16	188	−1.1 ± 1.6	0.5
Length (mm)						
2 wk	505 ± 21	257	508 ± 21	263	−3.2 ± 1.8	0.08
13 wk	596 ± 25	253	598 ± 26	257	−1.8 ± 2.3	0.4
52 wk	713 ± 30	242	715 ± 34	254	−1.9 ± 2.9	0.5
Weight (kg)						
2 wk	3.3 ± 0.5	257	3.3 ± 0.5	263	−0.02 ± 0.04	0.6
13 wk	5.8 ± 0.8	254	5.8 ± 0.8	259	0.02 ± 0.07	0.7
52 wk	7.9 ± 1.1	243	7.9 ± 1.0	254	0.04 ± 0.09	0.7
Head circumference (mm)						
2 wk	354 ± 14	256	355 ± 14	262	−0.3 ± 1.2	0.8
13 wk	399 ± 15	249	400 ± 13	257	−0.02 ± 1.2	0.9
52 wk	443 ± 15	242	444 ± 14	254	−0.49 ± 1.3	0.7
Triceps skinfold thickness (mm)						
2 wk	57 ± 13	257	57 ± 12	263	−0.1 ± 1.1	0.9
13 wk	77 ± 16	251	76 ± 16	258	0.5 ± 1.4	0.7
52 wk	72 ± 15	243	70 ± 16	252	2.1 ± 1.4	0.1
MUAC*^5^* (mm)						
2 wk	102 ± 9	257	101 ± 9	263	0.4 ± 0.8	0.6
13 wk	129 ± 11	254	128 ± 12	259	0.3 ± 1.0	0.7
52 wk	134 ± 13	242	133 ± 12	253	1.6 ± 1.1	0.1

1 Mother-infant pairs at delivery: *n* = 260 in the calcium group, and *n* = 265 in the placebo group. Statistical analysis was conducted by ANOVA or ANCOVA with Scheffé’s post hoc tests to determine differences according to maternal calcium or placebo group. Effects on growth velocity were tested by using repeated-measures ANOVA or ANCOVA as appropriate and performed with the use of hierarchical linear models that included participant identifier and sex (nested by group), time point, and birth order (parity of the mother; nulliparous compared with parous).

2 All values are means ± SDs.

3 All values are differences ± SEMs.

4 Assessments were conducted within 5 d of delivery.

5 MUAC, midupper arm circumference.

Hierarchical repeated-measures ANOVA with the subject (nested by intervention group) and time point by using data from 2, 13, and 52 wk showed significant increases over time within individuals but no significant differences between calcium and placebo groups at any time point or any significant intervention group-by–time point interaction for any variable.

Maternal postpartum BP and anthropometric measures at 2, 13, and 52 wk postpartum are shown in **Table 4**. There were significant decreases in SBP and DBP between 2 and 13 wk in the placebo group, and significant decreases in SBP, DBP, and weight between 2 and 52 wk and 13 and 52 wk both the calcium and placebo groups.

**TABLE 4:** Maternal blood pressure and anthropometric measurements in the first year postpartum by group allocation*^1^*

Time point and measurement	Calcium group*^2^*	*n*	Placebo group*^2^*	*n*	Calcium compared with placebo*^3^*	*P*	Calcium compared with placebo*^4^*	*P*
SBP (mm Hg)								
2 wk	107.7 ± 12.0	258	110.0 ± 15.2	262	−2.2 ± 1.2	0.07	−1.5 ± 0.9	0.1
13 wk	107.1 ± 10.4	250	107.8 ± 12.2^a^	259	−0.6 ± 1.0	0.5	−0.2 ± 0.8	0.8
52 wk	105.6 ± 9.1^a,b^	244	106.2 ± 11.0^a,b^	256	−0.5 ± 0.9	0.5	−0.14 ± 0.74	0.8
DBP (mm Hg)								
2 wk	64.4 ± 10.8	258	66.4 ± 12.1	262	−1.9 ± 1.0	0.05	−2.2 ± 1.4	0.1
13 wk	63.5 ± 10.8	249	64.5 ± 11.5^a^	259	−1.0 ± 1.0	0.3	−0.7 ± 1.5	0.6
52 wk	62.0 ± 9.0^a,b^	243	62.5 ± 9.5^a,b^	256	−0.5 ± 0.8	0.5	0.63 ± 1.23	0.6
Weight (kg)								
2 wk	53.9 ± 7.1	256	53.9 ± 6.9	263	0.05 ± 0.6	0.9	0.0 ± 0.5	0.9
13 wk	53.8 ± 7.4	253	53.6 ± 7.2	259	0.19 ± 0.6	0.8	0.11 ± 0.55	0.8
52 wk	52.6 ± 7.7^a,b^	244	52.7 ± 7.7^a,b^	256	−0.2 ± 0.7	0.8	0.86 ± 0.67	0.2
BMI (kg/m^2^)								
2 wk	21.0 ± 2.3	256	21.0 ± 2.4	263	0.1 ± 0.2	0.7	—	—
13 wk	21.0 ± 2.4	253	20.9 ± 2.6	259	0.1 ± 0.2	0.6	—	—
52 wk	20.6 ± 2.7	244	20.6 ± 2.7	256	0.0 ± 0.2	0.9	—	—

1 Mother-infant pairs at delivery: *n* = 260 in the calcium group, and *n* = 265 in the placebo group. Statistical analysis was performed by using Student's paired *t* tests and conditional multiple regression analysis with Linear Model software in the DataDesk version 6.3.1 program (Data Description Inc). ^a^Significantly different from 2 wk; ^b^significantly different from 13 wk. DBP, diastolic blood pressure; SBP, systolic blood pressure.

2 All values are means ± SDs.

3 All values are differences ± SEMs.

4 All values are percentages of change ± SEMs from 20 wk of gestation.

There were no significant differences between groups at any time point, except for a lower SBP (−2.2 ± 1.2 mm Hg; *P* = 0.07) and significantly lower DBP of a similar magnitude (−1.9 ± 1.0 mm Hg; *P* = 0.05) in the calcium group at 2 wk. There were no significant differences between groups in changes from P20. Significant positive predictors of SBP were weight at 2, 13 and 52 wk, height at 2 and 13 wk, and being nulliparous at P20 at 13 wk. Significant positive predictors of DBP were weight and height at 2 and 13 wk and nulliparity at P20 and season of P20 measurement at 52 wk. Significant positive predictors of weight were season of P20 measurement at 2, 13, and 52 wk, height and parity at 2 and 13 wk, and clinic area at 52 wk.

## DISCUSSION

This trial showed that an increase in calcium intake of 1500 mg/d during the second half of pregnancy in women accustomed to a very low calcium intake did not significantly affect BP in pregnancy or during 12 mo postpartum. The incidence of PIH [DBP ≥90 mm Hg or an increase of >15 or >30 mm Hg in DBP and SBP, respectively (2)] was ∼10%, with no difference between groups. Compliance was high, as indicated by direct observation, tablet counts, the measured change in urinary calcium, and the difference in urinary calcium between groups at P36. There are plausible explanations why our results differed from those of other trials.

The multicenter WHO calcium supplementation trial (8325 nulliparous women in Argentina, Egypt, India, Peru, South Africa, and Vietnam) used the same calcium dose and tablet composition and a similar protocol to those in our trial. There was no effect on preeclampsia, but severe PIH and eclampsia was significantly lower in the calcium group (18). Our trial could not be powered to examine preeclampsia or eclampsia because the population of The Gambia is too small to have generated sufficient incidence rates for these outcomes. Our trial was powered to detect between-group differences in SDP and DBP of 2.4%, which are physiologically plausible and would have shown a biologically significant effect of calcium supplementation. Such differences at a population level are significant; a reduction of BP by 2–3 mm Hg would greatly reduce associated morbidity (25).

In most other trials that have shown significant effects of calcium supplementation on BP, a low dietary calcium intake was defined as <600 or <900 mg Ca/d (2). In many trials, intake was not directly assessed in participants before and/or during supplementation but in similar groups or on the basis of population data. In a subset of 125 women in our trial, mean dietary calcium intake at P20 was 345 and 363 mg/d in calcium and placebo groups, respectively (32). We have no reason to believe that calcium and other nutrient intakes were different in the entire group. In rural Gambia, dietary calcium intake is very low throughout life (19, 42), and as we have reported, it is likely that the population is adapted to their very low calcium intake (31, 43). Thus, potential mechanisms of action of additional calcium on reducing BP such as via a reduction in PTH and consequent effects on vasodilation (2) may not apply. However, our findings also differed from those of 2 supplementation studies in Indian women [both 2000 mg compared with placebo (16, 17)] whose baseline calcium intakes were similar to those in our trial and in which significant reductions in the incidence of PIH and preeclampsia and SBP and DBP were reported. A possible reason for the discordance with other studies was that the comparability of the original randomly assigned groups may have been compromised by the exclusion of 20.7% of women from the final analysis.

Risk factors for PIH include overweight and obesity before, and high weight gains during, pregnancy. In the WHO trial, BMI was >30 in 17% of women (18). In one of the Indian studies, BMI ranged from 15 to 46 (16). In our trial, BMI was ≤20, 20–25, 25–30, and ≥30 in 28%, 64%, 7%, and 1% of women, respectively. Other trials have been conducted in settings where typical gestational weight gains were ∼12 kg and often >20 kg. In our trial, the mean weight gain from P20 to P36 was 4.2 kg, which was consistent with previous observations in this population (37, 44). Marked seasonal influences (because of physical activity, food availability, and infectious disease) on pregnancy weight gain, birth weight, and infant growth have also been described (30, 37). However, we showed no effect of the season on maternal weight gain, birth weight, or baseline BP, which possibly reflected secular trends in increased year-round food availability.

Participants in other trials (2) had higher BP at baseline than did participants in our trial (∼5 mm Hg for both SBP and DBP). Thus, the underlying prevalence of hypertension and, therefore, risk of PIH may have been lower in our trial. Information about smoking and drinking habits associated with raised BP was not collected because women of reproductive age in this population do not typically use tobacco or drink alcohol. At the time our trial was conducted, the prevalence of hypertension in The Gambia was greater in urban than rural areas, and age-related increases in BP were not apparent in women <45 y old (23–25). The remote rural farming area, mean BMI in the normal range, and very high levels of physical activity (45–47) may also partly explain why women may have been at lower risk of PIH. The prevalence of raised BP (>140/90 mm Hg) in women >25 y old is 24.8% globally, 35.5% in the WHO Africa region, and 34.2% in The Gambia (48). However, The Gambia is undergoing a rapid transition, and secular trends have indicated reductions in physical activity, an increasing prevalence of overweight and obesity, and comorbidities (49), and thus, risks of PIH may be different in the future.

Many calcium supplementation trials have focused on nulliparous and/or primiparous women because they are at higher risk of PIH (39). In our trial, 14% of participants were nulliparous, and 11% of participants were primiparous. However, a change in partner has also been associated with higher risk of PIH (50, 51). In rural Gambia, it is common for a woman to have children by different fathers because of early widowhood, divorce, and remarriage. Therefore, risk of PIH may not be confined to nulliparous or primiparous women, and it might be expected that the incidence of PIH would be higher. Marital data were not collected, and thus, this issue could not be explored.

It is possible that more cases of perinatal loss in the placebo group were related to hypertension, and their exclusion reduced the mean BP measurement in the placebo compared with calcium groups. This possibility was tested in a secondary analysis that included all women with a singleton pregnancy for whom a P36 BP measurement was available. ITT models showed no significant difference between calcium (*n* = 276) and placebo (*n* = 277) groups in the change in SBP at P36 compared with P20 (mean ± SEM percentage of change: −0.58 ± 0.63 mm Hg; *P* = 0.4) or DBP (mean ± SEM percentage of change: −0.15 ± 1.10 mm Hg; *P* = 0.9).

The WHO trial and our trial measured DBP to the fifth Korotkoff sound by using manual and automated sphygmomanometers, respectively. The Dinamap 8100 has been used successfully in large national surveys in the United Kingdom and, at the time of the trial, was regarded as the instrument of choice for research that involved different observers. Although concerns have since been expressed about an underestimation of DBP (52, 53), this was unlikely to have affected the results. Participants were randomly assigned, acted as their own controls, and measurements in both groups would have been potentially affected to the same extent.

Postpartum, there was no effect of supplementation on maternal BP, in contrast to effects on bone mineral status and calcium metabolism (31). There was also no effect on weight change during pregnancy or postpartum, which provided no evidence to support the proposed role of calcium in weight regulation (54) and was consistent with the conclusions of a recent review (55).

Potential adverse consequences for the offspring of low maternal calcium intake include poor fetal growth, low birth weight, and poor bone mineralization (55). We showed no effect of maternal calcium supplementation on any measures of growth at birth or during infancy, which confirmed our earlier findings in the subset (32) and was consistent with a review that concluded that maternal calcium supplementation has no significant effect on infant length or head circumference and only a small and clinically insignificant effect (65 g) on birth weight (55, 56). The possibility of longer-term effects on BP, growth, and bone development could not be excluded, and studies are underway to investigate this prospect.

The nutritional status of this rural Gambian population has been characterized previously (57). Energy restriction is common in pregnant and lactating women, and poor intakes of vitamins and other dietary factors have been recorded. It is possible that a limited supply of energy or nutrients prevented the anticipated response to the additional calcium. Although dietary intakes were not directly assessed in all participants, we had no reason to believe that these differed between intervention groups and obscured an effect of the calcium supplement.

In conclusion, in this randomized, placebo-controlled trial in more than 600 rural pregnant Gambian women with very low calcium intakes and which used robust methods, highly standardized techniques, and had very high compliance, there was no significant effect of calcium supplementation on maternal BP or infant growth. Differences between this trial and others may not be due to calcium availability but, instead, because the women were adapted to a low dietary calcium intake and/or obesity, high gestational weight gain, high underlying BP, tobacco use, alcohol consumption, and sedentary lifestyles were rare.
